# Blending gestation and lactation diets during the transition period reduces energy mobilization by sows in late gestation, with no impact on subsequent lactation performance

**DOI:** 10.1093/tas/txae096

**Published:** 2024-06-19

**Authors:** N Gregory, L Huber

**Affiliations:** Department of Animal Biosciences, University of Guelph, Guelph, ON, Canada N1G 2W1; Department of Animal Biosciences, University of Guelph, Guelph, ON, Canada N1G 2W1

**Keywords:** gilts, multiparous sows, subsequent lactation performance, transition feeding program

## Abstract

Fifty-three gilts and fifty-three multiparous (**MP**) sows were used to evaluate a blended feeding program using gestation and lactation diets during the transition period on changes in sow back fat (**BF**) depth and BW, blood metabolites, and litter growth performance in the subsequent lactation period. A 2 × 2 factorial experimental design was generated including the factors of parity and feeding program. The MP sows and gilts were assigned to one of two feeding programs on day 104 ± 1 of gestation: 1) 2 kg/d of a standard lactation diet until farrowing when sows received step-up access to the lactation diet until ad libitum access was given on day 4 of lactation (**CON**) and 2) a dynamic blend of standard gestation and lactation diets that met estimated daily requirements for standardized ileal digestible Lys and net energy according to the NRC (2012) until day 4 of lactation where sows were provided ad libitum access to the lactation diet (**TRAN**). Litters were standardized to 13 ± 1 piglets within 24-h of birth. In gestation, ADFI was greatest for TRAN-MP sows (interaction; *P* < 0.05), with greater ADFI for TRAN versus CON sows (main effect; 2.95 vs. 2.13 ± 0.08 kg; *P *< 0.05). Feeding program did not influence ADFI in lactation, but MP sows had greater ADFI versus gilts (main effect; 5.96 vs. 4.47 ± 0.28 kg; *P *< 0.001). Immediately after farrowing, TRAN sows had greater BW and BF vs. CON sows, regardless of parity (main effect; 224.1 vs. 215.4 ± 4.1 kg and 17.3 vs. 16.2 ± 0.4 mm, respectively; *P* < 0.05). At weaning, no feeding program-related differences were observed for BW or BF, but MP sows had thicker BF compared to gilts (main effect; 14.4 vs. 13.4 ± 0.5 mm; *P* < 0.05). The TRAN-MP sows had heavier piglets at birth compared to all other groups (interaction; *P *< 0.05) and MP sows had greater litter birth weight and average piglet BW at birth versus gilts (main effect; *P* < 0.05). No effect of feeding program was observed for piglet BW at weaning. On lactation day 1, serum beta-hydroxybutyric acid and non-esterified fatty acid concentrations were lower for TRAN compared to CON sows (main effect; 12.0 vs. 19.4 ± 7.8 mmol/L and 0.35 vs. 0.57 ± 0.10 mmol/L, respectively; *P* < 0.05) and serum glucose concentration was greater for TRAN compared to CON sows (main effect; 4.41 vs. 3.88 ± 0.22 mmol/L; *P* < 0.05), but these differences were no longer detectable at weaning. Therefore, a simple transition feeding program using a blend of a standard gestation and lactation diets reduced energy mobilization by sows in late gestation, with no impact on subsequent lactation performance.

## Teaser

A simple transition feeding program using a blend of gestation and lactation diets reduced energy mobilization by sows in late gestation, with no impact on subsequent lactation performance.

## INTRODUCTION

Proper feeding practices for sows during the reproductive cycle are essential to maximize sow lactation and reproductive performance. The transition period, recently defined as the last 5 to 7 d of gestation and the first 3 to 5 d of lactation, embodies significant changes in nutrient and energy demands to support fetal growth, mammary development, colostrum, and milk production (NRC, 2012; Feyera and Theil, 2017; reviewed by Theil et al., 2022), and includes major changes in feed intake and diet composition. Indeed, estimated energy and Lys requirements increase by 8% and 25%, respectively, in the final 20 d of gestation (NRC, 2012), with 17% of the estimated Lys requirement partitioned toward mammary development during the final 12 d of gestation (Feyera and Theil, 2017). Compared to day 104 of gestation, energy and Lys requirements increase by 228% and 338%, respectively, by peak lactation (day 17; NRC, 2012; Feyera and Theil, 2017; as reviewed by Tokach et al., 2019). When nutrient requirements are not met via dietary supply in gestation and/or lactation, the sow will mobilize energy and protein pools to meet the requirements for the products of conceptus and milk production (Dourmad et al., 2008). If the mobilization of maternal tissues is substantial, milk production and subsequent reproductive and litter performance are negatively affected (Strathe et al., 2017). Therefore, the changes in energy and nutrient demands during the transition period require careful consideration in order to maximize milk production and reproductive performance of the sow.

In practice, there are a variety of feeding strategies used in the swine industry during the transition period that are implemented based on sow flow and feed management constraints (e.g., number of feed lines available) within individual facilities (Theil, 2015). Often, sows are fed restricted amounts of a gestation diet (low energy and nutrient density) prior to movement into the farrowing house and restricted amounts of a lactation diet (high energy and nutrient density) until farrowing. Post-farrowing, a step-up to ad libitum or semi-ad libitum access to the lactation diet occurs. Such a feeding strategy does not mirror the changes in daily energy and nutrient requirements of the sow in the peripartum period. A dynamic blend of gestation and lactation diets, however, could better match estimated amino acid (Lys) and energy requirements, without requiring additional feed bins for specialized transition diets.

It was hypothesized that better meeting the estimated (daily) Lys and energy requirements during the transition period would improve lactation performance by supporting litter growth and minimizing maternal tissue mobilization. The objective of the study was to evaluate the effect of a specialized feed blending curve to match estimated daily standardized ileal digestible (**SID**) Lys and net energy (**NE**) requirements during the transition period on litter characteristics at birth and subsequent lactation performance in gilts and multiparous (**MP**) sows.

## MATERIALS AND METHODS

### Animals and Housing

The study was conducted at the Arkell Swine Research Station (Guelph, ON, Canada). All experimental protocols were approved by the University of Guelph Animal Care Committee (AUP 4172) and followed the Canadian Council of Animal Care Guidelines (CCAC, 2009). About 53 primiparous sows (gilts) and 53 MP sows (81 Yorkshire × Landrace and 25 Yorkshire) were recruited on day 104 ± 1 of gestation over five consecutive breeding batches (blocks). Between days 104 and 110 of gestation, sows were individually housed in gestation stalls and then were moved to farrowing crates equipped with a heat mat in the creep area. Within 24 h after birth, litters were standardized to 13 ± 1 piglets via cross fostering within treatments. Piglets were not provided with creep feed to allow body weight gain to reflect sow milk production.

### Feeding Programs

**Figure 1.**
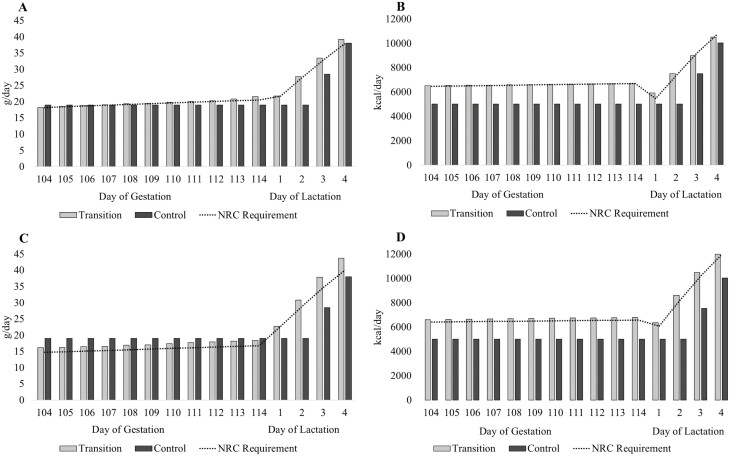
NE and SID Lys provided on each day of the transition period (day 104 of gestation until day 4 of lactation) for the transition feeding program (light gray bars) versus the control feeding program (dark gray bars) with projected requirements based on NRC (2012) recommendations (dotted line). (A) gilt SID lysine curve, (B) gilt NE curve, (C) multiparous sow (average parity 2.9 ± 0.1) SID lysine curve, (D) multiparous sow (average parity 2.9 ± 0.1) NE curve.

On day 104 ± 1 of gestation, gilts and MP sows (average parity: 2.9 ± 0.1) were assigned to one of two feeding programs in a 2 × 2 factorial design (*n* = 26 or 27) using the factors of parity and feeding program, accounting for initial BW and genetics. The two feeding programs were as follows: 1) 2 kg of a standard lactation diet provided per day until farrowing (lactation day 0) and then step-up access to the lactation diet until ad libitum access was given on day 4 of lactation (**CON**) and 2) a dynamic blend of a standard gestation and lactation diets that met estimated daily requirements for SID Lys and NE until day 4 of lactation where sows were then provided ad libitum access to the lactation diet (**TRAN**; Table 1). The transition feeding programs were designed using the NRC (2012) gestating sow and lactating sow models to estimate daily SID Lys and NE requirements within parity. Sow performance inputs used for the models were based on previous experimental data from the research station. Requirements during gestation for gilts were calculated using an anticipated BW at breeding of 151 kg and a protein deposition target of 105 g/d. For the MP sows, an average of parity 3 was used with an anticipated BW at breeding of 208 kg and a protein deposition target of 80 g/d. Both gilts and MP sows had gestation lengths set to 114, anticipated litter size of 13.5, and anticipated piglet birth weight of 1.40 kg. The lactation requirements were calculated using BW after farrowing of 195 and 220 kg and piglet average daily gains of 227 and 256 g over a 21-d lactation period for gilts and MP sows, respectively. The reader is directed to Fig. 1 for a visual representation of estimated daily NE and SID Lys requirements for gilts and MP sows compared to the NE and SID Lys supplied by each feeding program. Between gestation days 104 and 110, sows were hand-fed the allotted feed divided into two meals per day; upon entry to the farrowing rooms (day 110 of gestation), feed was provided via an automated feeding system with the capacity to blend two diets (Gestal Quatro Pro, JYGS Technologies, St-Lambert-de-Lauzon, QC, Canada). Beyond day 4 of lactation, sows received ad libitum access to feed via trigger manipulation within the feeder between 0500 and 2300 hours. Sows were weaned on day 20 ± 1 of lactation, which was the end of the experimental period.

**Table 1.** Diet composition and nutrient contents of diets used for the transition feeding programs (as-fed basis)

Item	Gestation^1^	Lactation^2^
Ingredient composition, %		
Corn	43.3	50.5
Soybean meal	14.5	28.2
Barley	32.0	—
Wheat	—	14.0
Wheat shorts	5.0	—
Fat, animal–vegetable blend	1.5	3.0
Limestone	1.3	1.5
Mono-calcium phosphate	1.3	1.6
Sodium chloride	0.4	0.4
Vitamin and mineral premix^3^	0.6	0.6
l-Lys-HCl	—	0.1
l-Thr	0.1	0.1
Total	100.0	100.0
Calculated Nutrient composition		
Net energy, Kcal/kg	2419	2507
Crude protein, %	15.00	19.79
SID^4^ Lys, %	0.58	0.95
Calcium, %	0.83	0.98
Phosphorus, %	0.66	0.74
Analyzed nutrient composition %		
Crude protein	15.27	18.69
Calcium	0.80	0.92
Phosphorus	0.60	0.65
Lys, %	0.68 (0.69)^5^	1.01 (1.09)

^1^Gestation diet was used in the transition feeding programs between day 104 of gestation until day 4 of lactation and was blended in daily varying proportions (from 72% at day 104 to 5% at day 4 of lactation for gilts and 97% at day 4 of gestation to 10% at day 4 of lactation for MP sows) with the lactation diet.

^2^Lactation diet was used for the control feeding program between day 104 of gestation until day 4 of lactation and was provided ad libitum to all sows thereafter.

^3^Treatment diets provided per kg of premix: vitamin A, 2,000,000 IU as retinyl acetate; vitamin D3, 200,000 IU as cholecalciferol; vitamin E, 8,000 IU as dl-α-tocopherol acetate; vitamin K, 500 mg as menadione; pantothenic acid, 3,000 mg; riboflavin, 1,000 mg; choline, 100,000 mg; folic acid, 400 mg; niacin, 5,000 mg; thiamin, 300 mg; pyridoxine, 300 mg; vitamin B_12_, 5 mg; biotin, 40 mg; Cu, 3,000 mg from CuSO_4_ × 5H_2_O; Fe, 20,000 mg from FeSO_4_; Mn, 4,000 mg from MnSO_4_; Zn, 21,000 mg from ZnSO_4_; Se, 60 mg from Na_2_SeO_2_ and I, 100 mg from KI (DSM Nutritional Products Canada Inc., Ayr, ON, Canada).

^4^Standardized ileal digestible.

^5^Calculated values are shown in parentheses.

### Experimental Observations

Sow BW measurements were collected on days 104 and 110 of gestation, within 24 h of farrowing, and at weaning. While sows were housed in gestation stalls, feed refusals were collected daily to calculate feed intake. Once sows were moved to the farrowing room, feed intake was recorded daily using the feeding system software (JYGS Technologies). Backfat (**BF**) depth was measured using an ultrasound (Honda Electric Co. Ltd, Toyohashi, Aichi, Japan) at the P2 position on day 104 of gestation, at farrowing (day 0), and weaning. Litter characteristics (i.e., number born alive, stillborns, mummies, and litter birthweight) were recorded within 24 h of farrowing. Individual piglet BW was measured at birth, on day 1 of lactation, and at weaning.

After a 12-h fast, blood samples were collected via orbital sinus puncture from a subset of sows in each parity-feeding program combination on day 1 of lactation and at weaning: CON-gilt (*n* = 12), CON-MP (*n* = 13), TRAN-gilt (*n* = 12), and TRAN-MP (*n* = 12). Blood was collected in a 10 mL untreated serum tube (BD vacutainer, Franklin Lakes, NJ), left to clot for 60 min, and centrifuged at room temperature at 1,500 × *g* for 15 min. The serum was subsampled and stored at −20 °C until further analysis. On day 18 ± 1 of lactation, piglets were removed from these same sows and placed in an adjacent pen equipped with a heat source for 1 h. Sows were then injected IM with 1 mL of oxytocin (20 USP/mL; Oxyto-Sure, Vetoquinol, Cambridge, ON) to induce milk letdown. About 75 mL of milk was collected from each front and rear gland, pooled, and then stored at −20 °C until further analysis.

### Laboratory Analyses

Diets were sampled at the beginning of each block, samples were combined within diet and were further subsampled for analyses of crude protein (AOAC, 2005; method 968.06) and calcium and phosphorus using inductively couple plasma-optical emission spectrometry (AOAC, 2005; method 985.01; Agrifood Laboratories, Guelph, ON, Canada). Lysine in feed was analyzed via ultra-performance liquid chromatography via acid hydrolysis. Derivatization was completed using an AccQ-Tag Ultra derivatization kit (Method 994.12; AOAC, 2005; Waters Corporation, Milford, MA). The obtained amino acid peak areas were compared to known standard and analyzed using Waters Empower 2 Software (Waters Corporation). Sow serum samples were analyzed by University of Guelph Animal Health Laboratories (Guelph, ON, Canada) using a Conas 600 c501 biochemistry analyzer (Rocher Diagnosis, Laval, QC, Canada) to determine concentrations of beta-hydroxybutyric acid (**BHBA**), non-esterified fatty acids (**NEFA**), glucose, and urea. Milk samples were thawed and vortexed prior to subsampling 30 mL of milk, which was freeze-dried to determine dry matter content. Freeze-dried samples were then used to determine crude fat via high-temperature solvent extraction (Ankom, XT29 Fat Analyzer, Macedon, NY; AOCS, 2017; Official Procedure Am 5-04) and nitrogen content via combustion analysis (LECO-FP 828 analyzer, LECO Instruments Ltd., Mississauga, ON, Canada). Nitrogen values were multiplied by 6.38 to calculate crude protein content.

### Statistics Analyses

Statistical analyses were completed using the Proc GLIMMIX function of SAS using sow (or litter for the offspring outcomes) as the experimental unit. For sow and piglet performance, blood metabolites, and milk composition outcomes, the model included the fixed effects of treatment (feeding program; TRAN or CON), parity (gilt or MP sow), and the interaction of treatment and parity. The random effects of block and sow within block and a repeated measure statement were included. Litter size was used as a covariate for piglet BW outcomes. Log transformations were completed for serum BHBA and NEFA concentrations; back transformations were completed to present the results. Mean comparisons were conducted using the Tukey Kramer post-hoc test when the interaction was significant. Probability (*P*) values less than 0.05 were considered significant.

## RESULTS

Calculated and analyzed nutrient contents were comparable for the gestation and lactation diets (Table 1). Three sows were removed from the study due to complications around farrowing (2 TRAN-MP and 1 TRAN-gilt) and two of the recruited sows were not pregnant (1 CON-gilt and 1 TRAN-gilt); data from these animals were not included in the statistical analyses.

### Sow Growth Performance

Sow BW was influenced by parity throughout the entirety of the study with MP sows always being heavier than gilts (*P* < 0.05; Table 2). No treatment or interaction effects were observed at day 104 or 110 of gestation or at weaning for sow BW. However, immediately after farrowing TRAN sows were heavier than CON sows, regardless of parity (*P* < 0.05). No treatment or interaction effects were observed for sow ADG, but ADG was greater for MP sows than for gilts between days 104 and 110 of gestation (*P *< 0.05) and MP lost less BW per day compared to gilts during lactation (*P* < 0.05).

**Table 2.** Growth performance of gilts and multiparous (MP) sows that received a control or transition feeding program in the peripartum period

	Control^1^		Transition			*P* value^2^		
Item	Gilt	MP	Gilt	MP	SEM^3^	Trmt	Parity	Trmt * Parity
No.^4^	25	26	25	25				
Body weight, kg								
Gestation day 104	208.4	251.9	209.2	254.5	7.2	0.695	<0.001	0.827
Gestation day 110	217.5	256.2	218.5	262.7	6.2	0.348	<0.001	0.495
Lactation day 0^5^	192.8	237.9	198.1	250.2	4.1	0.042	<0.001	0.409
Weaning	174.4	229.2	179.4	233.6	4.7	0.267	<0.001	0.959
Average daily gain, kg								
Gestation days 104 to 110	1.52	0.67	1.57	1.42	0.43	0.057	0.026	0.092
Lactation	−0.92	−0.37	−0.89	−0.80	0.26	0.093	0.013	0.056
Back fat, mm								
Gestation day 104	17.2	16.9	17.0	17.9	0.5	0.294	0.597	0.134
Lactation day 0	16.7	15.7	17.1	17.4	0.4	0.015	0.421	0.127
Weaning	13.2	13.8	13.5	14.9	0.5	0.121	0.037	0.410
Back fat change, mm								
Gestation day 104 to lactation day 0	−0.48	−1.24	0.11	−0.63	0.37	0.048	0.018	0.961
Lactation	−3.50	−1.88	−3.46	−2.48	0.42	0.448	<0.001	0.393
Average daily feed intake, kg								
Gestation	2.08^c^	2.18^c^	2.75^b^	3.14^a^	0.08	<0.001	<0.001	0.026
Lactation	4.40	6.12	4.54	5.80	0.28	0.531	<0.001	0.124

^1^Control sows received 2 kg of the lactation diet from day 104 of gestation until farrowing. Transition sows received a blend of gestation and lactation diets to meet estimated daily SID Lys and NE requirements based on NRC recommendations (2012) fed between day 104 of gestation and day 4 of lactation. Ad libitum access to the lactation diet was provided in both feeding programs after day 4 of lactation.

^2^Probability values for the main effects of feeding program (Trmt), parity, and the interaction between feeding program and parity (Trmt * Parity).

^3^Maximum value for standard error of means.

^4^Number of animals evaluated; multiparous sow average parity: 2.9 ± 0.1.

^5^Lactation day zero was the day of farrowing; sows were weaned on day 20 ± 1 of lactation.

^a,b,c^Means without a common superscript within a row differ (*P* < 0.05).

Sow BF was not influenced by the main effect of treatment, parity, or the interaction of treatment and parity on day 104 of gestation, and no interaction effects for sow BF were observed at farrowing or at weaning. However, at farrowing TRAN sows had thicker BF compared to CON sows, regardless of parity (*P* < 0.05; Table 2). At weaning MP sows had thicker BF compared to gilts (*P* < 0.05), however difference in BF between treatments was no longer present. Between day 104 of gestation and farrowing, TRAN sows lost less BF compared to CON sows (*P* < 0.05) and MP sows lost more BF compared to gilts (*P* < 0.05). During lactation, MP sows lost less BF compared to gilts (*P *< 0.001), but dietary treatment effects were no longer observed.

The ADFI during late gestation was influenced by treatment, parity, and the interaction between treatment and parity (*P *< 0.05; Table 2) due to the nature of the feeding programs. The TRAN-MP sows consumed the most feed compared to all other groups (*P *< 0.05), TRAN gilts consumed more feed than CON gilts and MP sows (*P *< 0.05), the latter two of which were not different from one another. During lactation, ADFI was not influenced by feeding program or the interaction between feeding program and parity, but was greater for MP sows versus gilts (*P* < 0.001).

### Litter Characteristics and Piglet Performance

Litter characteristics (numbers born alive, stillborn, and mummies) were not influenced by treatment, parity, or the interaction between treatment and parity, but litter birth weight was greater for MP sows versus gilts (*P* < 0.001; Table 3). Piglet BW at birth and on day 1 were influenced by the interaction between treatment and parity, such that piglets from MP sows fed TRAN were heavier than piglets from gilts fed TRAN, gilts fed CON, and MP sows fed CON (at birth only; *P* < 0.05). By weaning, the interaction between treatment and parity no longer influenced piglet BW, but piglet ADG over the lactation period and piglet BW at weaning were greater for MP sows versus gilts (*P *< 0.001), regardless of feeding program.

**Table 3.** Litter characteristics and offspring growth performance throughout lactation for gilts and multiparous (MP) sows that received a control or transition feeding program in the peripartum period

	Control^1^		Transition			*P* value^2^		
Item	Gilt	MP	Gilt	MP	SEM^3^	Trmt	Parity	Trmt * Parity
No.^4^	25	26	25	25				
Litter characteristics								
Born alive, no.	12.6	13.0	11.9	12.2	0.6	0.174	0.562	0.881
Stillborn, no.	1.1	1.4	0.9	0.8	0.3	0.112	0.780	0.439
Mummies, no.	0.3	0.4	0.5	0.4	0.2	0.611	0.892	0.536
Litter birth weight, kg	18.18	20.77	16.50	19.93	0.73	0.084	<0.001	0.558
Litter size at birth^5^	13.3	13.4	13.4	13.4	0.3	0.434	0.587	0.630
Litter size at wean^6^	12.7	11.6	12.5	12.1	0.3	0.679	0.005	0.159
Piglet body weight, kg								
Birth	1.34^b^	1.44^b^	1.32^b^	1.62^a^	0.05	0.086	<0.001	0.034
Day 1	1.50^b^	1.56^ab^	1.43^b^	1.71^a^	0.04	0.421	<0.001	0.030
Weaning^6^	5.99	6.46	5.76	6.27	0.19	0.161	<0.001	0.892
Piglet overall average daily gain, kg	0.23	0.25	0.22	0.24	0.01	0.228	<0.001	0.779

^1^Control sows received 2 kg of the lactation diet from day 104 of gestation until farrowing. Transition sows received a blend of gestation and lactation diets to meet estimated daily SID Lys and NE requirements based on NRC recommendations (2012) fed between day 104 of gestation and day 4 of lactation. Ad libitum access to the lactation diet was provided in both feeding programs after day 4 of lactation.

^2^Probability values for the main effects of feeding program (Trmt), parity, and the interaction between feeding program and parity (Trmt * Parity).

^3^Maximum value for standard error of means.

^4^Number of litters evaluated; multiparous sow average parity: 2.9 ± 0.1.

^5^Litter size after standardization.

^6^Weaning occurred on day 20 ± 1 of lactation.

^a,b^Means without a common superscript within a row differ (*P* < 0.05).

### Serum Metabolites

On lactation day 1, serum BHBA and NEFA concentrations were lower for TRAN versus CON sows, regardless of parity (*P* < 0.05; Table 4) but by weaning there was no influence of treatment, parity, or the interaction of treatment and parity. On lactation day 1, serum urea was influenced by the interaction between treatment and parity (*P *< 0.05) but no differences were detected among treatment means. By weaning, serum urea was only influenced by parity (*P *< 0.001) where MP sows had greater serum urea concentrations than gilts, regardless of feeding program. On lactation day 1, serum glucose was greater for TRAN versus CON sows, regardless of parity (*P *< 0.05). At weaning, serum glucose was influenced only by the main effect of parity (*P *< 0.05) where MP sows had lower serum glucose concentrations compared to gilts, regardless of feeding program.

**Table 4.** Serum metabolite concentrations for gilts and multiparous (MP) sows that received a control or transition feeding program in the peripartum period

	Control^1^		Transition			*P* value^2^		
Item	Gilt	MP	Gilt	MP	SEM^3^	Trmt	Parity	Trmt * Parity
No.^4^	12	13	12	12				
BHBA, mMol/L								
Lactation day 1	23.7	15.1	10.5	13.4	7.8	0.014	0.609	0.066
Weaning^5^	50.0	62.1	52.5	55.2	15.6	0.790	0.335	0.518
NEFA, mMol/L								
Lactation day 1	0.63	0.50	0.32	0.37	0.10	0.011	0.622	0.303
Weaning	1.07	1.66	1.30	1.25	0.33	0.654	0.198	0.105
Urea, mMol/L								
Lactation day 1	3.20	2.98	2.99	3.98	0.34	0.175	0.196	0.042
Weaning	4.21	5.50	4.47	5.37	0.42	0.803	<0.001	0.480
Glucose, mMol/L								
Lactation day 1	3.73	4.02	4.64	4.18	0.22	0.029	0.696	0.126
Weaning	2.63	1.78	2.69	2.27	0.35	0.249	0.012	0.375

^1^Control sows received 2 kg of the lactation diet from day 104 of gestation until farrowing. Transition sows received a blend of gestation and lactation diets to meet estimated daily SID Lys and NE requirements based on NRC recommendations (2012) fed between day 104 of gestation and day 4 of lactation. Ad libitum access to the lactation diet was provided in both feeding programs after day 4 of lactation.

^2^Probability values for the main effects of feeding program (Trmt), parity, and the interaction between feeding program and parity (Trmt * Parity).

^3^Maximum value for standard error of means.

^4^Number of animals evaluated; multiparous sow average parity: 2.9 ± 0.1.

^5^Weaning occurred on day 20 ± 1 of lactation.

### Milk Chemical Composition

Milk dry matter content was not influenced by the main effects of treatment, parity, or the interaction between treatment and parity (Table 5). Milk crude protein concentration was greater for MP sows than gilts, regardless of feeding program (*P *< 0.05), and crude fat concentration was greater for TRAN versus CON sows, regardless of parity (*P *< 0.05).

**Table 5.** Chemical composition of milk on day 18 of lactation for gilts and multiparous (MP) sows that received a control or transition feeding program in the peripartum period (as-is basis)

	Control^1^		Transition			*P* value^2^		
Item	Gilt	MP	Gilt	MP	SEM^3^	Trmt	Parity	Trmt * Parity
No.^4^	12	12	11	12				
Dry matter, %	18.46	18.90	19.06	19.10	0.55	0.413	0.636	0.683
Crude protein, %	5.02	5.16	4.74	5.32	0.10	0.623	0.025	0.165
Crude fat, %	7.27	7.01	8.00	8.09	0.53	0.039	0.856	0.691

^1^Control sows received 2 kg of the lactation diet from day 104 of gestation until farrowing. Transition sows received a blend of gestation and lactation diets to meet estimated daily SID Lys and NE requirements based on NRC recommendations (2012) fed between day 104 of gestation and day 4 of lactation. Ad libitum access to the lactation diet was provided in both feeding programs after day 4 of lactation.

^2^Probability values for the main effects of feeding program (Trmt), parity, and the interaction between feeding program and parity (Trmt * Parity).

^3^Maximum value for standard error of means.

^4^Number of animals evaluated; multiparous sow average parity: 2.9 ± 0.1.

## DISCUSSION

The purpose of the current study was to assess a simple transition feeding program that blended gestation and lactation diets to match estimated daily SID Lys and NE requirements for gilts and sows on litter characteristics at birth and subsequent lactation performance. In the current study, sows on the TRAN feeding program were better able to maintain BF throughout late gestation compared to sows on the CON feeding program, while gilts that received the TRAN program were the only group to gain BF depth between day 104 of gestation and farrowing. Moreover, immediately after farrowing, TRAN sows had greater maternal body weight vs. sows on the CON feeding program, regardless of parity. Since there were no feeding program effects on litter birthweight, it can be suggested that BW gain during late gestation occurred in maternal or mammary tissues rather than to support additional fetal protein deposition when sows received the TRAN feeding program. In addition, fasted serum BHBA and NEFA concentrations were lower and glucose concentrations were greater on day 1 of lactation for sows that received the TRAN feeding program compared to sows that received the CON feeding program, regardless of parity. Since BHBA is produced during the catabolism of fatty acids and ketogenic amino acids, elevated serum concentrations indicate that the CON sows were utilizing ketogenic versus glucogenic forms of energy in addition to the breakdown of body fat stores (Theil et al., 2013). It can also be inferred that the TRAN sows were able to utilize energy from the diet and glucogenic precursors from the liver around farrowing (Theil et al., 2022). Elevated NEFA concentrations are also an indicator of a catabolic state and the mobilization of maternal body fat stores to meet the increased energy demands of the products of conceptus, colostrum, and milk production during the transition period (Dourmad et al., 2008; Peltoniemi and Oliviero, 2015; Theil, 2015). Therefore, the TRAN feeding program was better able to meet the energy (and Lys) requirements for maintenance, growth of the products of conceptus, and maternal energy (and protein) deposition, indicating that traditional transition feeding programs with restricted intakes of gestation and lactation diets do not meet the energy, and possibly Lys requirements, of sows and gilts during late gestation.

In the current study, sows on the TRAN program received more feed per day than sows on the CON program during late gestation, per the study design, while both gilts and sows on the CON feeding program received the same amount of feed per day. High feed (energy) intake during late gestation relative to energy requirements can result in excessive body lipid deposition, which often reduces milk production and piglet growth during the subsequent lactation period due to reductions in lactation feed intake (Dourmad et al., 2008; Hansen et al., 2012; Strathe et al., 2017). Obviously, an electronic feeding system was required for the TRAN program in the current study, which represents a significant financial investment for producers. Nonetheless, the increasing popularity of electronic feeding systems for sows, particularly alongside the implementation of loose sow housing, provides an avenue for producers to execute a blended feeding program using existing gestation and lactation diets, without the logistics of creating and delivering a dedicated transition diet. The ADFI observed during lactation period in the current study was also lower than projected for both gilts and MP sows (i.e., 4.47 vs. 5.95 kg for gilts and 5.96 vs. 6.61 kg for MP sows; NRC, 2012) but was not influenced by transition feeding program. Moreover, piglet BW at weaning and piglet ADG throughout lactation were also not influenced by transition feeding program. Since creep feed was not provided to the piglets, the lack of difference between the two treatments for litter growth implies there was no difference in milk production between the feeding programs. Bruun et al. (2023) found a linear improvement in piglet ADG and BW at weaning when sows were fed increasing levels of feed, and therefore energy (23.3 to 64.5 MJ ME/d), from day 108 of gestation until farrowing. Conversely, others demonstrated limited effects of various transition feeding programs on subsequent milk production by the sow (e.g., Decaluwé et al., 2014; Gourley et al., 2020), indicating that both sows and gilts have significant capacity to maintain milk production regardless of daily nutrient and energy supply in the transition period.

In the current study, sows that received the TRAN program had greater milk fat contents versus CON on day 18 of lactation. Though there were no feeding program-related differences in BF loss during the lactation period, and sows that received the TRAN program may have partitioned more lipid toward milk. Greater milk fat content has been shown to support greater piglet growth rates during the lactation period (Amdi et al., 2013; Rosero et al., 2015), but in the current study, no feeding program effects were observed for piglet growth rate. It is possible that the composition of piglet BW gain was altered (i.e., with greater lipid deposition rates) since milk protein content did not increase along with milk fat in the TRAN-fed sows, though assessment of offspring chemical body composition was beyond the scope of the current study. Previous work has shown that increased body lipid content in young pigs promotes accelerated protein deposition rates in the energy-dependent phase and when the dietary supply of amino acids is not limiting protein deposition (Totafurno et al., 2019), which could reduce the post-weaning growth lag (Gondret et al., 2020). Therefore, future work should also examine weaning and post-weaning changes in piglet body composition and growth performance as carry-over effects from the maternal transition and lactation feeding programs.

In the current study, many differences were observed between MP sows and gilts. For example, MP sows had greater loss of BF depth by the end of gestation compared to gilts, despite having no differences in BF depth at day 104 of gestation. Energy requirements are estimated to increase by 1.64 × between days 104 and 115 of gestation (Feyera and Theil, 2017), with the increased loss of BF for MP sows implying that the MP sows were undersupplied energy and utilized fat stores to meet energy requirements for maintenance and reproduction, regardless of feeding program. Sow daily energy requirements were estimated with the NRC (2012) gestating and lactating sow models using parameters from previous experimental data collected at the research station. However, the MP sows recruited in the current study were between 20 and 30 kg heavier than expected, therefore sows received approximately 300 kcal/day below NE requirement estimates. In contrast, MP sows lost less BW and BF during lactation compared to gilts, which also aligns with MP sows exhibiting greater ADFI capacity as has been shown many times previously (e.g., as reviewed by Eissen et al., 2000; Strathe et al., 2017). Moreover, MP sows had larger piglets at birth and piglets achieved greater ADG throughout the lactation period compared to the offspring of gilts, indicating that MP sows had greater milk production than gilts. Milk is produced via secretory cells present in mammary parenchymal tissue, and greater parenchymal tissue mass has been correlated to greater milk production (Nielsen et al., 2001). Farmer et al. (2023) found that MP sows have 40% greater mammary parenchymal tissue mass at day 110 of gestation compared to gilts, which likely influenced milk production capacity in the current study, regardless of feeding program.

Finally, in the current study, the milk of MP sows had greater crude protein concentration compared to that of gilts on day 18 of lactation. Previous studies comparing the chemical composition of milk from MP sows versus gilts found no differences in protein concentration among parities (Craig et al., 2019). In the current study, it is possible that greater feed intake by sows resulted in greater amino acid availability for milk protein production, despite the observed greater (fasted) plasma urea concentrations, indicative of catabolism of excess and/or unbalanced amino acids.

## CONCLUSION

In conclusion, blending gestation and lactation diets to meet estimated SID Lys and NE requirements during the transition period reduced maternal energy mobilization prior to farrowing with no lasting effect on lactation performance. Therefore, a simplified transition feeding program that blends standard gestation and lactation diets could be used on commercial farms with the capacity to blend feed sows, without the need to formulate and manage an additional “transition” diet.
